# Elucidating Pharmacological Mechanisms of Natural Medicines by Biclustering Analysis of the Gene Expression Profile: A Case Study on Curcumin and Si-Wu-Tang

**DOI:** 10.3390/ijms16010510

**Published:** 2014-12-29

**Authors:** Yuan Quan, Bin Li, You-Min Sun, Hong-Yu Zhang

**Affiliations:** 1 Agricultural Bioinformatics Key Laboratory of Hubei Province, College of Informatics, Huazhong Agricultural University, Wuhan 430070, China; E-Mails: qyuan@webmail.hzau.edu.cn (Y.Q.); skylib777@gmail.com (B.L.); 2 School of Municipal and Environmental Engineering, Shandong Jianzhu University, Jinan 250101, China; E-Mail: sunym@sdu.edu.cn

**Keywords:** natural medicines, pharmacology, biclustering analysis, curcumin, Si-Wu-Tang

## Abstract

Natural medicines have attracted wide attention in recent years. It is of great significance to clarify the pharmacological mechanisms of natural medicines. In prior studies, we established a method for elucidating pharmacological mechanisms of natural products contained in connectivity map (cMap), in terms of module profiles of gene expression in chemical treatments. In this study, we explore whether this methodology is applicable to dissecting the pharmacological mechanisms of natural medicines beyond the agents contained in cMap. First, the gene expression profiles of curcumin (a typical isolated natural medicine) and Si-Wu-Tang (a classic traditional Chinese medicine formula) treatments were merged with those of cMap-derived 1309 agents, respectively. Then, a biclustering analysis was performed using FABIA method to identify gene modules. The biological functions of gene modules provide preliminary insights into pharmacological mechanisms of both natural medicines. The module profile can be characterized by a binary vector, which allowed us to compare the expression profiles of natural medicines with those of cMap-derived agents. Accordingly, we predicted a series of pharmacological effects for curcumin and Si-Wu-Tang by the indications of cMap-covered drugs. Most predictions were supported by experimental observations, suggesting the potential use of this method in natural medicine dissection.

## 1. Introduction

It is widely accepted that natural medicines have made great contributions to safeguarding human health, and have provided a rich source of modern drugs [1]. Thus, it is of great significance to elucidate the therapeutic mechanisms of natural medicines. However, this is a grand challenge, because natural medicines usually consist of complex components and hit multiple targets with relatively weak affinity. In the current omics era, various omic technologies have been used to elucidate the therapeutic mechanisms of natural medicines, in which DNA microarray is of special interest [2].

DNA microarray offers a relatively cheap and easily handle facility to systematically characterize the gene expression profiles for cells or tissues, which can be used to identify the gene expression variations in response to chemical treatment. Recently, microarray analysis showed great potential in elucidating mode of action for natural medicines [3,4,5,6]. However, the prior methods for gene expression profile analysis normally use a small part of signature genes, which lose a lot of useful information. Since gene expression signatures for different biological activities group into different modules [7,8], we speculated that the pharmacological mechanisms of natural medicines may be more efficiently analyzed by considering the module organization of gene expression profiles.

In our recent studies, we used biclustering analysis to generate biological-relevant modules for connectivity map (cMap)-derived gene expression profiles upon chemical treatments [9], and elucidated the polypharmacological mechanisms for 20 polyphenols through comparing their gene module profiles with those of approved drugs [10]. This preliminary success stimulated our interest to explore whether this methodology is applicable to clarifying the pharmacological mechanisms of natural medicines beyond the agents contained in cMap.

In the present study, we attempt to elucidate the medicinal effects of two well-known natural medicines, *i.e.*, curcumin (a typical isolated natural medicine [11,12]) and Si-Wu-Tang (a classic traditional Chinese medicine formula, consisting of *Radix Angelicae sinensis*, *Radix Rehmanniae Preparata*, *Radix Paeoniae Alba* and *Rhizoma Chuanxiong* [13]), by gene module analysis. First, the microarray data of curcumin and Si-Wu-Tang treatments were merged with those of 1309 chemical treatments in cMap, respectively. Then, a biclustering analysis was performed using FABIA (factor analysis for bicluster acquisition) method, which allowed us to elucidate the complex pharmacological mechanisms of curcumin and Si-Wu-Tang in terms of gene modules.

## 2. Results and Discussion

First, the gene expression profiles of human monocytes (U937 cells) treated with curcumin (1 μM) for 18 h (GSE10896) [14] were combined with the expression profiles of cMap-derived 1309 agents [15], which results in a matrix of 22,215 rows (probes) and 1310 columns (agents). FABIA 2.2.2 software [16] was employed to search K biclusters of the matrix, where K (number of biclusters) was set to 50. The sparseness factor was set to 0.1 and the iteration number was set to 20,000. When K ≥ 49, superfluous biclusters information contents were close to zero, indicating that biclusters contained all the information of the matrix. Bicluster 1 involves the richest information and bicluster 49 involves the poorest (Figure S1). The 49 biclusters consisted of 7084 probes and were ordered according to their information contents. It should bear in mind that in the present study, a gene module is exactly a bicluster, because a module is not only a set of genes, but also linked with a set of agents. Thus, each agent in 1310 samples has a gene module profile that can be characterized by a 49-dimensional binary vector, with 1 or 0 representing the presence or not of the module (Table S1).

Second, by using a similar procedure, we processed gene expression data of MCF-7 cell line treated with Si-Wu-Tang at concentration of 2.56 mg/mL (GSE23610) [4]. The 22,215 × 1310 matrix, derived from the combination of Si-Wu-Tang and cMap data, was grouped into 53 biclusters by FABIA algorithm (Figure S1), which consist of 6120 probes. The gene module profile for each agent characterized by a 53-dimensional binary vector was presented in Table S2 .

**Figure 1:**
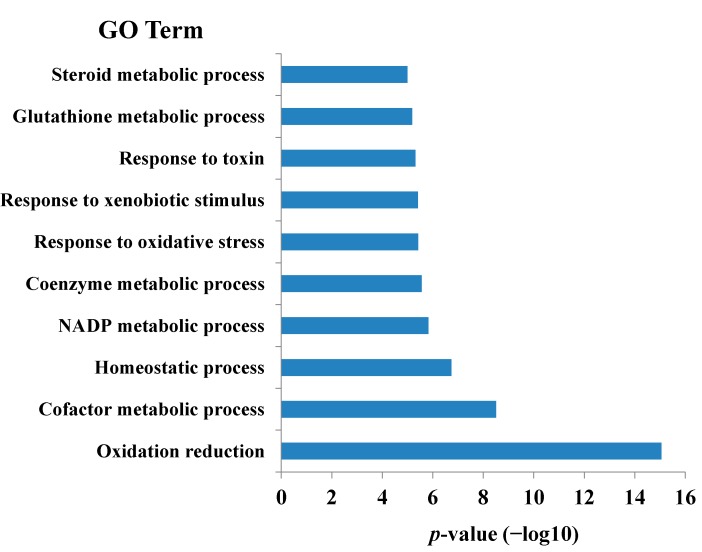
The enriched GO function of module 27 of cMap-Si-Wu-Tang dataset, with *p*-values adjusted by False Discovery Rate calculation using Benjamini-Hochberg method [24].

**Figure 2:**
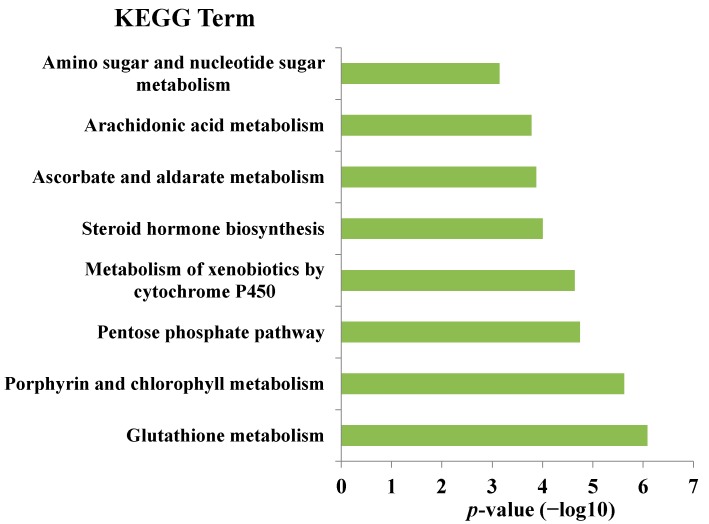
The enriched KEGG function of module 27 of cMap-Si-Wu-Tang dataset, with *p*-values adjusted by False Discovery Rate calculation using Benjamini-Hochberg method [24].

The biological functions were enriched for each module by the records in GO and KEGG pathways (DAVID) [17]. For the cMap-curcumin dataset, 49 modules have enriched GO functions, with 47 having significant KEGG functions (Table S3). For the cMap-Si-Wu-Tang dataset, 53 modules have enriched GO functions, with 51 having significant KEGG functions (Table S4). For both datasets, GO functions and KEGG pathway annotations match well with each other, illustrating the functional consistence of the modules. Thus, we can get some preliminary insights into the medicinal effects of curcumin and Si-Wu-Tang according to the module functions. For instance, module 27 of Si-Wu-Tang dataset is tightly associated with oxidative reduction according to the GO and KEGG (Figure 1 and Figure 2). This module contains the antioxidant genes controlled by nuclear factor (erythroid-derived 2)-like 2 (Nrf2), such as *GPX2*, *FTH1*, *GCLM*, *GCLC*, *NQO1*, *HMOX1*, *GSR* and *PRDX1* [18]. It is well known that the Nrf2-mediated Keap1-Nrf2-ARE pathway is the most important cellular defense pathway against oxidative stress in human body [19]. Therefore, module 27 can be defined as an antioxidant module. Indeed, some known antioxidants, such as ascorbic acid, ebselen, tanespimycin, 1,4-chrysenequinone, menadione, tetroquinone [9], are included in this module. Because Si-Wu-Tang is also involved in this module, it can be inferred that Si-Wu-Tang has antioxidant function, well consistent with the experimental observation [4].

A similar analysis indicates that module 8 of curcumin dataset is associated with oxidative reduction (Table S3). However, curcumin is not included in this module, suggesting that curcumin cannot activate Nrf2 to generate antioxidant effect at low concentration (1 μM), which agrees well with the observation by Meja *et al.* [14]. This conclusion is further supported by quantum chemical calculations.

**Figure 3:**
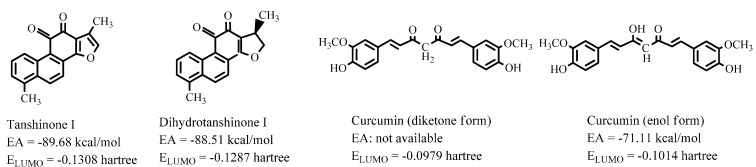
Electron affinity (EA) and energy level of the lowest unoccupied molecular orbital (E_LUMO_) of tanshinones and curcumin calculated at B3LYP/6-31+G(d) level. The data of tanshinones are from [20].

In a recent study, we demonstrated that the parameters characterizing electron-abstracting capacity, such as electron affinity (EA) and energy level of the lowest unoccupied molecular orbital (E_LUMO_), can measure the Nrf2-activating potential of natural antioxidants [20], because electrophilic modification of cysteine residues in Keap1 is a major mechanism for Nrf2 activation [21]. Thus, we calculated EA and E_LUMO_ for curcumin by a density functional theory (DFT) method and compared the results with those of tanshinones, which are strong Nrf2-activators even at low concentration (2.5 μM) [22]. As shown in Figure 3, the EA or E_LUMO_ of curcumin (for diketone and enol forms) are considerably higher than those of tanshinone I and dihydrotanshinone I, which implies that curcumin is a much weaker electrophilic agent than the tanshinones. In combination with the poor bioavailability of curcumin [23], it is concluded that this well-known pigment is of trivial value as an *in vivo* antioxidant.

A more thorough elucidation of pharmacological mechanisms for curcumin and Si-Wu-Tang can be performed by gene module profile analysis. Since the module profile is characterized by a binary vector, we can calculate Tanimoto coefficient (*TC*) for the module profiles of each agent pairs according to Equation (1) [10]:

(1) $$TC=\frac{N_{\text{AB}}}{N_{\text{A}}+N_{\text{B}}-N_{\text{AB}}}$$

where *N*_A_ and *N*_B_ are the number of bits set for gene module profiles of agents A and B, respectively, and *N*_AB_ is the set bits that A and B have in common. A high Tanimoto coefficient means agent pairs have similar biological effects. In such a case, if one of the agent pairs has drug indication information, we can infer the medicinal effects of the other [10].

**Figure 4:**
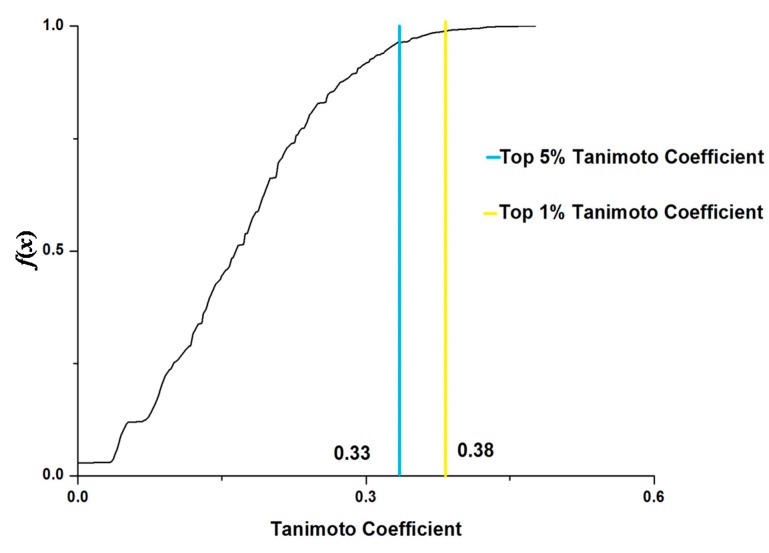
Cumulative frequency (*f*(*x*)) of pairwise Tanimoto coefficients for cMap-curcumin dataset.

For the cMap-curcumin dataset, a total of 857,395 pairwise Tanimoto coefficients were calculated for the 1310 agents. The top 1% Tanimoto coefficients are higher than 0.38 (Figure 4). There are 19 agents that have similar gene module profiles with curcumin (with Tanimoto coefficients >0.38). By searching DrugBank [25] and ChemBank [26], we found that 7 agents have clear drug indications (Table 1), in which 2 (esculin and clobetasol) have an anti-inflammatory effect, 3 (amantadine, oxolinic acid and lomefloxacin) have an anti-infective effect, and 2 (sulpiride and amantadine) are for neurological disorders. Accordingly, we infer that anti-inflammatory, anti-infective, and neurological regulation are the most fundamental biological effects of curcumin, which indeed agrees well with the experimental observations [11,12].

**Figure 5:**
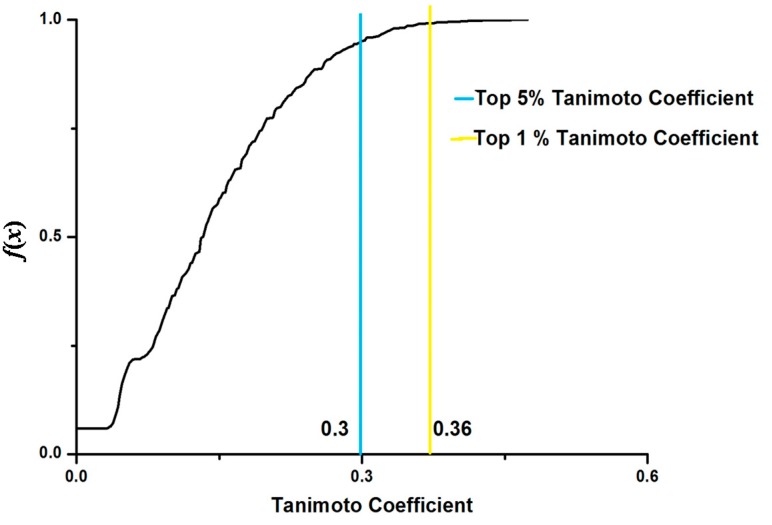
Cumulative frequency (*f*(*x*)) of pairwise Tanimoto coefficients for cMap-Si-Wu-Tang dataset.

A similar analysis was performed for cMap-Si-Wu-Tang dataset. The top 1% Tanimoto coefficient threshold is 0.36 (Figure 5). There are 17 agents similar to Si-Wu-Tang, in terms of gene module profile. From DrugBank [25] and ChemBank [26], we found that 10 agents have therapeutic uses (Table 2), which include 3 anti-neoplastic agents (carmustine, lomustine and diethylstilbestrol), 3 anti-infective or anti-inflammatory agents (clioquinol, celastrol and mometasone), 2 vasodilatation agents (withaferin A and nifedipine), and 1 tranquilizer (spiperone). It is noteworthy that diethylstilbestrol also has an estrogen-like effect, which is the most important efficacy of Si-Wu-Tang [4]. Besides, the anti-neoplastic, antibacterial, vasodilatation and sedative effects are consistent with the actual effectiveness of Si-Wu-Tang [4,27].

**Table 1:** Predicted similar drugs to curcumin.

Drugs	Tanimoto Coefficients	Therapeutic Uses
Esculin ^a^	0.48	Anti-inflammatory
Clobetasol ^b^	0.42	Anti-inflammatory Agents, Corticosteroids, Topical, Glucocorticoids
Iodixanol ^b^	0.41	Contrast Media
Sulpiride ^b^	0.38	Antidepressants, Antidepressive Agents, Second-generation, Antipsychotic Agents, Antipsychotics
Amantadine ^b^	0.38	Analgesics, Non-narcotic, Antiparkinson Agents, Anti-viral Agents, Dopamine Agents
Oxolinic acid ^a^	0.38	Anti-bacterial
Lomefloxacin ^b^	0.38	Anti-infective Agents, Anti-infective Agents, Urinary, Antitubercular Agents, Photosensitizing Agents

^a^ The Therapeutic Uses of drugs from ChemBank [25]; ^b^ The Therapeutic Uses of drugs from DrugBank [26].

**Table 2:** Predicted similar drugs to Si-Wu-Tang.

Drugs	Tanimoto Coefficients	Therapeutic Uses
Clioquinol ^a^	0.41	Anti-infective Agent
Carmustine ^b^	0.41	Antineoplastic Agents, Antineoplastic Agents, Alkylating
Celastrol ^a^	0.40	Anti-bacterial, Anti-proliferative
Spiperone ^a^	0.38	Tranquilizer
Mometasone ^b^	0.38	Anti-allergic Agents, Anti-inflammatory Agents
Lomustine ^b^	0.37	Antineoplastic Agents, Antineoplastic Agents, Alkylating
Withaferin A ^b^	0.36	Calcium Channel Blockers, Dihydropyridines, Tocolytic Agents, Vasodilator Agents
Nifedipine ^b^	0.36	Vasodilator Agents
Diethylstilbestrol ^b^	0.36	Antineoplastic Agents, Hormonal, Carcinogens, Estrogens, Non-steroidal
Chlorzoxazone ^b^	0.36	Muscle Relaxants, Central

^a^ The Therapeutic Uses of drugs from ChemBank [25]; ^b^ The Therapeutic Uses of drugs from DrugBank [26].

## 3. Experimental Section

### 3.1. Data Preprocessing

The gene expression data of curcumin treatment (GSE10896, including 3 treatment chips and 3 control chips) [14] and Si-Wu-Tang treatment (GSE23610, including 3 treatment chips and 3 control chips) [4] were downloaded from NCBI [28]. The gene expression data for five cultured human cell lines treated with 1309 agents were downloaded from connectivity map (cMap) [15], which consist of 6100 treatment chips and 956 control chips.

The raw data were first normalized by Robust Multi-array Average expression measure [9]. For cMap-derived agents, the expression values were usually determined under different conditions. Therefore, the median of the values were used to represent the expression profile. Then, the expression profiles treated by curcumin and Si-Wu-Tang were combined with the cMap-derived expression profiles, respectively. This resulted in a matrix of 22,215 rows (probes) and 1310 columns (including 1309 cMap-derived agents and curcumin/Si-Wu-Tang). The data in each column were normalized using Equation (2):

(2) $$x_{ij}^{*}=\frac{x_{ij}-x_{j}}{s_{j}}$$

where *x_ij_* is the expression value in row *i* and column *j*, *x_j_* is the mean value of column *j* and *s_j_* is the standard deviation of column *j*.

### 3.2. Biclustering Analysis

Biclustering analysis was performed by FABIA 2.2.2 software [16]. The principle and detailed procedure can refer to previous publications [9,16].

### 3.3. Density Functional Theory Calculation

Density functional theory (DFT) method (RO)B3LYP/6-31+G(d) was employed to optimize the structures and determine the vibrational frequencies for curcumin and its anions in vacuum. Then, single-point electronic energies were calculated by (RO)B3LYP functional [29] at 6-311+G(2d,2p) level. Solvation (water) effect was calculated on the single-point level using the polarizable continuum model (PCM) of the self-consistent reaction field (SCRF) theory [30]. As a result, molecular energy (E) consists of (RO)B3LYP/6-311+G(2d,2p)-calculated total electronic energy and (RO)B3LYP/6-31+G(d)-derived zero point vibrational energy (ZPVE, scaled by a factor of 0.9805) [31]. According to the definition of EA [32], EA = E_a_ − E_p_, where E_a_ is the energy of curcumin anion, and E_p_ is the energy of parent curcumin. All of the quantum chemical calculations were accomplished by the Gaussian 03 program [33].

## 4. Conclusions

In our prior studies, we established a gene module-based method to elucidate the pharmacological mechanisms of natural products contained in cMap [10]. Now, we show that this method can be extended to the natural medicines beyond cMap-covered agents. Curcumin is one of most extensively studied natural products, and Si-Wu-Tang is a classic traditional Chinese medicine formula. Through combining the gene expression profiles of curcumin and Si-Wu-Tang treatments with those of cMap-contained 1309 chemical treatments, we performed a biclustering analysis on the synthetic data and identified gene modules. The biological functions of the modules allowed preliminary dissection for the pharmacological mechanisms of both natural medicines. The gene module profile comparison between the natural medicines and cMap-derived agents enabled us to annotate the medicinal effects of curcumin and Si-Wu-Tang by the known drug indications. It is intriguing to note that the predicted biological effects of both natural medicines are well supported by experimental observations. Considering the maturity and facility of DNA microarray technique, this methodology is expected to find wide applications in elucidating the complex pharmacological mechanisms of natural medicines, not just for isolated natural products but also for herb formulae. However, it should be kept in mind that because the present approach is based on cMap data, it is only applicable to exploring the pharmacological mechanisms of human drugs rather than antimicrobial agents. Besides, the effectiveness of this method may be weakened by the limited chemicals and cell lines recorded in cMap. Therefore, a comprehensive elucidation of pharmacological mechanisms of natural medicines should combine various methods to perform the analysis.

## Supplementary Materials

Supplementary materials can be found at http://www.mdpi.com/1422-0067/16/01/0510/s1.

## References

[1] Ji H.F., Li X.J., Zhang H.Y. (2009). Natural products and drug discovery. EMBO Rep..

[2] Quan Y., Wang Z.Y., Xiong M., Xiao Z.T., Zhang H.Y. (2014). Dissecting traditional Chinese medicines by omics and bioinformatics. Nat. Prod. Commun..

[3] Cheng H.M., Li C.C., Chen C.Y., Lo H.Y., Cheng W.Y., Lee C.H., Yang S.Z., Wu S.L., Hsiang C.Y., Ho T.Y. (2010). Application of bioactivity database of Chinese herbal medicine on the therapeutic prediction, drug development, and safety evaluation. J. Ethnopharmacol..

[4] Wen Z., Wang Z., Wang S., Ravula R., Yang L., Xu J., Wang C., Zuo Z., Chow M.S., Shi L. (2011). Discovery of molecular mechanisms of traditional Chinese medicinal formula Si-Wu-Tang using gene expression microarray and connectivity map. PLoS One.

[5] Lin W.C., Tan T.W. (1994). The role of gastric muscle relaxation in cytoprotection induced by San-Huang-Xie-Xin-Tang in rats. J. Ethnopharmacol..

[6] Cheng W.Y., Wu S.L., Hsiang C.Y., Li C.C., Lai T.Y., Lo H.Y., Shen W.S., Lee C.H., Chen J.C., Wu H.C. (2008). Relationship between San-Huang-Xie-Xin-Tang and its herbal components on the gene expression profiles in HepG2 cells. Am. J. Chin. Med..

[7] Suthram S., Dudley J.T., Chiang A.P., Chen R., Hastie T.J., Butte A.J. (2010). Network-based elucidation of human disease similarities reveals common functional modules enriched for pluripotent drug targets. PLoS Comput. Biol..

[8] Iskar M., Zeller G., Blattmann P., Campillos M., Kuhn M., Kaminska K.H., Runz H., Gavin A.C., Pepperkok R., van Noort V. (2013). Characterization of drug-induced transcriptional modules: Towards drug repositioning and functional understanding. Mol. Syst. Biol..

[9] Xiong M., Li B., Zhu Q., Wang Y.X., Zhang H.Y. (2013). Identification of transcription factors for drug-associated gene modules and biomedical implications. Bioinformatics.

[10] Li B., Xiong M., Zhang H.Y. (2014). Elucidating polypharmacological mechanisms of polyphenols by gene module profile analysis. Int. J. Mol. Sci..

[11] Prasad S., Gupta S.C., Tyagi A.K., Aggarwal B.B. (2014). Curcumin, a component of golden spice: From bedside to bench and back. Biotechnol. Adv..

[12] Strimpakos A.S., Sharma R.A. (2008). Curcumin: Preventive and therapeutic properties in laboratory studies and clinical trials. Antioxid. Redox Signal..

[13] Yeh L.L., Liu J.Y., Lin K.S., Liu Y.S., Chiou J.M., Liang K.Y., Tsai T.F., Wang L.H., Chen C.T., Huang C.Y. (2007). A randomised placebo-controlled trial of a traditional Chinese herbal formula in the treatment of primary dysmenorrhoea. PLoS One.

[14] Meja K.K., Rajendrasozhan S., Adenuga D., Biswas S.K., Sundar I.K., Spooner G., Marwick J.A., Chakravarty P., Fletcher D., Whittaker P. (2008). Curcumin restores corticosteroid function in monocytes exposed to oxidants by maintaining HDAC2. Am. J. Respir. Cell Mol. Biol..

[15] Lamb J., Crawford E.D., Peck D., Modell J.W., Blat I.C., Wrobel M.J., Lerner J., Brunet J.P., Subramanian A., Ross K.N. (2006). The Connectivity Map: using gene-expression signatures to connect small molecules, genes, and disease. Science.

[16] Hochreiter S., Bodenhofer U., Heusel M., Mayr A., Mitterecker A., Kasim A., Khamiakova T., van Sanden S., Lin D., Talloen W. (2010). FABIA: Factor analysis for bicluster acquisition. Bioinformatics.

[17] Huang da, W., Sherman B.T., Lempicki R.A. (2009). Systematic and integrative analysis of large gene lists using DAVID bioinformatics resources. Nat. Protoc..

[18] Kaidery N.A., Banerjee R., Yang L., Smirnova N.A., Hushpulian D.M., Liby K.T., Williams C.R., Yamamoto M., Kensler T.W., Ratan R.R. (2013). Targeting Nrf2-mediated gene transcription by extremely potent synthetic triterpenoids attenuate dopaminergic neurotoxicity in the MPTP mouse model of Parkinson’s disease. Antioxid. Redox Signal..

[19] Kensler T.W., Wakabayashi N., Biswal S. (2006). Cell survival responses to environmental stresses via the Keap1-Nrf2-ARE pathway. Annu. Rev. Pharmacol. Toxicol..

[20] Sun Y.M., Xiao Z.T., Zhang H.Y. (2014). Structure-activity relationships of tanshinones in activating Nrf2. A DFT study and implications for multifunctional antioxidant discovery. Nat. Prod. Commun..

[21] Abiko Y., Miura T., Phuc B.H., Shinkai Y., Kumagai Y. (2011). Participation of covalent modification of Keap1 in the activation of Nrf2 by tert-butylbenzoquinone, an electrophilic metabolite of butylated hydroxyanisole. Toxicol. Appl. Pharmacol..

[22] Tao S., Zheng Y., Lau A., Jaramillo M.C., Chau B.T., Lantz R.C., Wong P.K., Wondrak G.T., Zhang D.D. (2013). Tanshinone I activates the Nrf2-dependent antioxidant response and protects against As(III)-induced lung inflammation *in vitro* and *in vivo*. Antioxid. Redox Signal..

[23] Sharma R.A., Euden S.A., Platton S.L., Cooke D.N., Shafayat A., Hewitt H.R., Marczylo T.H., Morgan B., Hemingway D., Plummer S.M. (2004). Phase I clinical trial of oral curcumin: Biomarkers of systemic activity and compliance. Clin. Cancer Res..

[24] Benjamini Y., Hochberg Y. (1995). Controlling the false discovery rate: A practical and powerful approach to multiple testing. J. R. Stat. Soc. B..

[25] Wishart D.S. (2008). DrugBank and its relevance to pharmacogenomics. Pharmacogenomics.

[26] Seiler K.P., George G.A., Happ M.P., Bodycombe N.E., Carrinski H.A., Norton S., Brudz S., Sullivan J.P., Muhlich J., Serrano M. (2008). ChemBank: A small-molecule screening and cheminformatics resource database. Nucleic Acids Res..

[27] Liang Q.D., Gao Y., Tan H.L., Guo P., Li Y.F., Zhou Z., Tan W., Ma Z.C., Ma B.P., Wang S.Q. (2006). Effects of four Si-Wu-Tang’s constituents and their combination on irradiated mice. Biol. Pharm. Bull..

[28] NCBI http://www.ncbi.nlm.nih.gov/.

[29] Lee C., Yang W., Parr R.G. (1988). Development of the Colle-Salvetti correlation energy formula into a functional of the electron density. Phys. Rev. B Condens. Matter.

[30] Miertuš S., Scrocco E., Tomasi J. (1981). Electrostatic interaction of a solute with a continuum. A direct utilization of ab initio molecular potentials for the prevision of solvent effects. Chem. Phys..

[31] Scott A.P., Radom L. (1996). Harmonic vibrational frequencies: an evaluation of Hartree Fock, Møller-Plesset, quadratic configuration interaction, density functional theory and semiempirical scale factors. J. Phys. Chem..

[32] Rienstra-Kiracofe J.C., Tschumper G.S., Schaefer H.F. (2002). Atomic and molecular electron affinities: Photoelectron experiments and theoretical computations. Chem. Rev..

[33] Frisch M.J., Trucks G.W., Schlegel H.B., Scuseria G.E., Robb M.A., Cheeseman J.R., Zakrzewski V.G., Montgomery J.A., Stratmann R.E., Burant J.C. (2004). GAUSSIAN 03.

